# Analysis of Safety and Efficacy of the Early Initiation of Antithrombotic Secondary Prevention in Patients Treated with Intravenous Thrombolysis for Acute Ischemic Stroke

**DOI:** 10.3390/jcm13092710

**Published:** 2024-05-05

**Authors:** Georgi Krastev, Miroslav Mako, Zuzana Števková, Romana Havranová, Kristína Andrášiková

**Affiliations:** 1 Clinic of Neurology, Faculty Hospital Trnava, A. Žarnova 11, 91701 Trnava, Slovakia; georgi.krastev@fntt.sk (G.K.); miroslav.mako@fntt.sk (M.M.); rhavranova@gmail.com (R.H.); kik.andrasikova@gmail.com (K.A.); 2 Clinic of Neurology in Trnava, Slovak Medical University, Limbová 12, 83303 Bratislava, Slovakia

**Keywords:** early antiplatelet therapy, alteplase, ischemic stroke, safety, effectiveness, early neurological deterioration

## Abstract

**Background and Objectives**: Current guidelines and the alteplase product insert recommend that antithrombotic therapy be avoided within 24 h of intravenous thrombolytic therapy with rt-PA in acute ischemic stroke. Therefore, the rate of stroke recurrence is unclear in terms of early neurological deterioration, which we could prevent with the early administration of antithrombotic therapy. We do not know the effect of early antithrombotic therapy after intravenous thrombolysis with rt-PA in acute stroke on the outcome in patients after 90 days either. **Design**: Prospective monocentric observational cohort study. **Methods**: Data were collected from consecutive patients treated with alteplase for acute ischemic stroke between January 2015 and January 2023. We examined functional outcome at 90 days, including the risk of symptomatic intracranial hemorrhage and mortality rate as safety indicators and stroke recurrence events in both early and standard antithrombotic therapy at 24 h after intravenous thrombolysis. **Results**: A total of 489 patients were included, of which 278 (56.9%) were men. Of these, 407 (83.2%) patients received early antithrombotic therapy. No symptomatic intracranial hemorrhage occurred in any participants. There was a significantly higher number of patients with an excellent outcome (mRS 0-1) in early antithrombotic treatment (211 (53.1%) versus 28 (34.6%) in standard antithrombotic treatment (*p* = 0.002, OR 0.47, 95% CI: 0.28–0.76). **Conclusions**: Early antithrombotic treatment after intravenous therapy in patients with acute ischemic stroke revealed no safety concerns compared with standard antithrombotic therapy and resulted in a significantly higher proportion of patients with an excellent functional outcome.

## 1. Introduction

Intravenous thrombolytic therapy (IVT) with alteplase has been the standard treatment for acute stroke since 1995 based on the results of the National Institute of Neurological Disorders and Stroke (NINDS) trial [1]. In this trial, the requirement not to administer anticoagulant and antithrombotic treatment (AT) 24 h after thrombolytic treatment appeared for the first time in the study protocol. This prohibition was later adopted and accepted, perhaps uncritically, in other clinical trials with a substantial benefit for the intravenous thrombolytic treatment of acute ischemic stroke (AIS), such as the European Cooperative Acute Stroke Study 3 (ECASS 3) in 2008 [2], but also, for example, in the guidelines for the treatment of AIS of the world’s professional organizations such as the American Stroke Association and the European Stroke Organisation [3,4].

**Figure 1:**
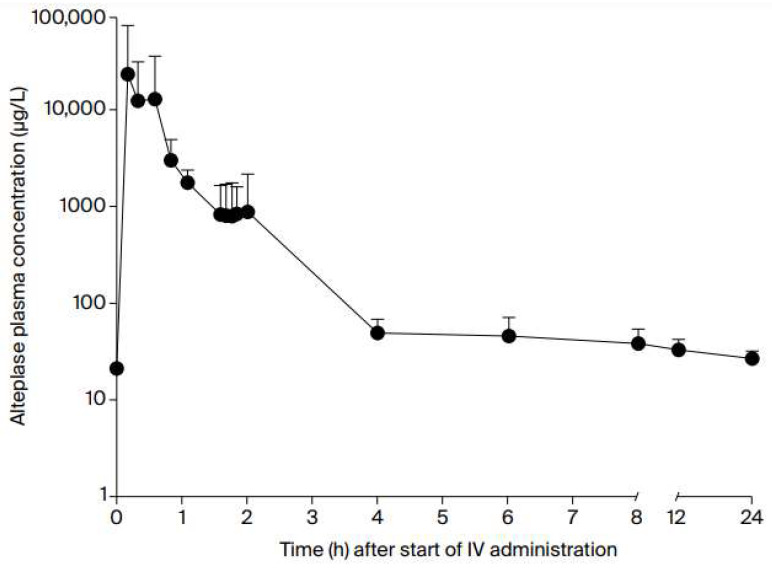
Serum levels of rt-PA after IVT administration [5].

Alteplase is a selective plasminogen activator with high affinity for fibrin on the clot surface, with extremely rapid pharmacodynamics. Already 10 min after the end of a 60 min infusion of alteplase in the treatment of AIS, 80% of the total amount of drug in the circulation is metabolized. Three to four hours after the start of alteplase administration, the fibrinolytic effect of the drug is negligible due to low plasma concentration [5], as shown in Figure 1. 

Therefore, it has not been considered that alteplase as a fibrinolytic may have a protective antithrombotic effect during the first 24 h. Thus, patients after IVT remain unprotected from a possible recurrence of stroke during this fragile time interval. Based on this fact, the prohibition of an early initiation of antithrombotic treatment (eAT) within 24 h after IVT is unreasonable and may have a significant effect on increasing the incidence of a recurrence of ischemic stroke and early neurological deterioration (END), and consequently also result in a worse outcome in patients after IVT.

Therefore, in this prospective monocentric observational cohort study, we tested the effect of using eAT after IVT for AIS outside the current guidelines for alteplase use (deviation from the thrombolytic protocol). We compared safety (the incidence of symptomatic intracranial hemorrhage (sICH) and the incidence of mortality within 90 days after IVT) and efficacy (the incidence of END and functional outcome 3 months after IVT) between the two groups.

## 2. Materials and Methods

We performed a monocentric analysis of prospectively collected clinical data of patients with acute ischemic stroke treated with intravenous rt-PA. Patients meeting criteria for IVT in our tertiary stroke center between January 2015 and January 2023 were evaluated.

We collected baseline data on age, sex, risk factors, pre-stroke disability, neurological symptom severity, blood pressure, serum glucose and fibrinogen levels. Furthermore, the presence of large or medium vessel occlusion was analyzed.

We analyzed data on treatment with mono AT or dual AT. We monitored the worsening of neurological status within the first 24 h after IVT, and between 24 h after IVT and 7 days or discharge from hospital, if the discharge was earlier than 7 days.

As safety markers, we collected data on the incidence of all sICHs on the follow-up brain CT scans after the start of AT and 3-month mortality.

We also analyzed the clinical outcomes of the patients after 3 months and the etiology of ischemic stroke.

Patient disability was evaluated using the modified Rankin scale (mRS) [6]. Patients with a good clinical outcome had mRS 0-2 and those with an excellent functional outcome had mRS 0 to 1. Neurological status was calculated according to the National Institutes of Health Stroke Scale (NIHSS) [7]. END was defined as an increase in the NIHSS score by four or more points compared to the initial NIHSS score within the first 24 h. We also analyzed a stricter criterion—an increase in the NIHSS score by one or more points compared to the initial NIHSS score within the first 24 h. We also monitored an increase in the NIHSS score by four or more points or by one or more points, respectively, compared to the NIHSS score 24 h after IVT and that between 24 h and 7 days or discharge, if it was earlier than 7 days.

The etiology of AIS was established according to the Trial of ORG 10172 in Acute Stroke Treatment (TOAST) classification after a diagnostic work-up [8]. Bleeding on a follow-up brain CT scan was evaluated as symptomatic based on the ECASS 3 criteria [2].

Patients were always examined by a neurologist experienced in stroke care. Blood samples were taken for the analysis of biochemical parameters (glycaemia, urea, creatinine, ionogram, osmolality and CRP), blood count evaluation and coagulation tests.

Imaging studies included non-contrast brain CT scans and CT angiography from the aortic arch to the vertex. Perfusion brain CT was performed in cases of unknown symptom onset. Patients were examined using a GE Revolution^©^ CT machine. They were administered Scanlux 370^©^ or Iomeron 400^©^ contrast material for the CTA and perfusion CT examinations. CT scans were evaluated by an experienced radiologist blinded to the clinical data.

IVT was administered in the emergency department at a dose of 0.90 mg/kg (10% bolus intravenously, and then the remaining 90% in a continuous intravenous infusion over the course of 60 min).

After the treatment, all patients were transferred to the stroke unit. A non-contrast CT scan was performed 6 to 16 h after IVT, or sooner in cases of neurological deterioration. If we did not find bleeding on the follow-up CT, the initiation of eAT was left to the stroke neurologist.

For the purpose of data processing, the patients were divided into two groups. In the first group were patients with eAT started earlier than 24 h after IVT. In the second group were patients whose treatment did not deviate from the standard protocol, where sAT was initiated 24 h after IVT. The above parameters were compared between the two groups.

We used oral aspirin (100 mg/day) and/or clopidogrel (75 mg/day), which could be preceded by a loading dose of 300 mg. The decision to initiate mono AT or dual AT and the administration of loading dose were left to the stroke neurologist.

Additional plain CT scans were performed in cases of neurological deterioration during hospitalization.

This study was approved by the Ethical Committee of Faculty Hospital, Trnava, Slovakia.

The Chi-square test was used to determine the existing differences in the frequencies of qualitative variables. Yates’ correction was used in cases where the frequencies were lower than 10. The normality of the data distribution of quantitative variables was assessed using the Kolmogorov–Smirnov test. Since the quantitative variables were from a non-normal distribution, the mean values were compared using a two-sample independent Wilcox test.

Statistical significance was determined by *p* ≤ 0.05. All data analysis was performed using SPSS statistical software, version 28.0.0.0.

## 3. Results

### 3.1. Baseline Data 

Overall, 1130 patients received rt-PA between 1 January 2015 and 31 January 2023 at our tertiary stroke center. Of these, we excluded 590 patients treated with mechanical thrombectomy (MT) and 51 patients with early intracranial hemorrhage after IVT before starting AT. The remaining 489 patients were assigned to eAT or sAT at the physician’s decision. We used dual AT in 366 (89.9%) patients in the eAT group and mono AT in the remaining 41 (10.1%).

There were 211 (43.1%) female patients in the study cohort and the median age was 71 years. There was no difference in the prevalence of DM and FA, while patients with sAT had more frequent AH (76.9% vs. 89.0%, *p* = 0.014). There was also no difference in pre-stroke disability assessed by means of mRS between the eAT and sAT groups. The median baseline NIHSS score was 7 points (0–26). Patients in the sAT group had a significantly worse baseline NIHSS score (7 vs. 8, *p* = 0.019). There was no difference in baseline values of BP and glycemia between the groups, while patients in the sAT group had significantly higher baseline values of fibrinogen. We did not observe a difference in the number of patients treated with ASA or clopidogrel before the index stroke. LVO or MeVO was identified in 147 (30.1%) patients, with no significant difference between the two investigated groups (Table 1).

### 3.2. Safety and Efficacy Data

In terms of efficacy, we monitored the incidence of END defined as an early (within 24 h) worsening of neurological symptoms by four or more NIHSS points compared to the baseline NIHSS score. In the entire cohort of patients, 32 (6.5%) patients had END. The difference in the incidence of END between the eAT and sAT groups was not significant, but END was more frequent in the sAT group. The difference was even more pronounced when we used a more stringent criterion for END, namely, a worsening of neurological symptoms by one or more NIHSS points compared to the baseline NIHSS score.

In the group of patients treated with IVT only, we found 48 (8.9%) with END (a worsening of neurological symptoms by four or more NIHSS points). After excluding patients with hemorrhage after IVT, the incidence of END was 6.5%. We found no statistical difference in the incidence of neurological deterioration between 24 h and 7 days or discharge (in case the patient was discharged within 7 days of hospital admission), assessed as a worsening of neurological symptomatology by four or more NIHSS points compared with the NIHSS at 24 h after IVT administration. However, again, there was a tendency for a higher incidence in the sAT group. Using a more stringent criterion, in the sAT group, we found a significantly more frequent occurrence of neurological deterioration between 24 h and 7 days or discharge, assessed as a worsening of neurological symptomatology by one or more NIHSS points compared with NIHSS at 24 h after IVT administration (15.9% vs. 6.4%, *p* = 0.004). END within 24 h was twice as high compared with neurological deterioration between 24 h and 7 days or discharge (6.5% vs. 3.5%) (Table 2).

**Figure 2:**
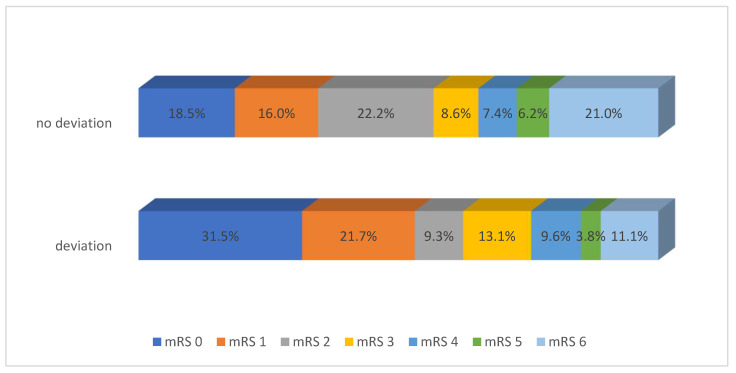
Three-month functional outcomes.

We found out 3-month mRS in 478 (97.8%) of the patients. None out of the 11 patients lost to follow died during the follow-up periods. After 3 months, patients in the eAT group were significantly more likely to have an excellent clinical outcome, assessed as mRS 0 to 1 (53.1% vs. 34.6%, *p* = 0.002). They were also significantly more likely to be free of any residual symptoms (mRS score 0, 31.5% vs. 18.5%, *p* = 0.019); Table 3, Figure 2.

As a safety precaution, we made the incidence of symptomatic hemorrhage after AT deployment our primary objective. After AT deployment, we did not observe neurological deterioration caused by intracranial hemorrhage in any patient in either group. Patients in the sAT group had a significantly higher risk of mortality (20.7% vs. 10.8%, *p* = 0.013) at 3 months after IVT (Table 2).

There were significantly more patients with cardioembolic etiology of AIS in the sAT group; we found no other significant differences in other types of etiology according to the TOAST criteria. (Table 4).

## 4. Discussion

The aim of our study was to determine the safety and efficacy of eAT compared with sAT in patients after IVT for AIS. From 1 January 2015 to 31 January 2023, we followed 489 patients assigned into two groups who received either eAT or sAT based on the clinician’s decision. 

Considering the complexity of the issue, there are few papers that have accurately determined the incidence of recurrent stroke and its causes in the early 24 h time period after IVT, as well as its prognostic significance for patient outcome. In a systemic review, Seners and Turco [9] adopted the definition of END as a worsening of neurological deficit by four or more NIHSS points. END occurs in 13.8% of patients after IVT according to the pooled incidence in their review, with intracranial hemorrhage being the cause in approximately 20% and malignant edema in a further 20% of the patients. However, in the majority of patients, the exact cause of END could not be identified. In both groups of patients, with and without IVT, the predictors associated with END during the first 24 h after acute stroke were baseline hyperglycemia, no prior aspirin use, prior transient ischemic attacks, proximal arterial occlusion and the presence of early ischemic changes on CT scans. The most consistent 24 h follow-up-associated factors were no recanalization or reocclusion of the vessel, large infarcts and intracranial hemorrhage. Finally, END was strongly predictive of poor outcome.

In a prospective multicenter observational cohort study from China [10] following patients after IVT, in which they defined END as a deterioration in NIHSS by two or more points, a 14.1% incidence of END within 24 h of admission was found. Age, body mass index, systolic blood pressure, baseline NIHSS, pre-stroke disability, a history of atrial fibrillation, diabetes mellitus, intracranial arterial stenosis and infarct location in the territory of the lenticulostriate artery were identified as independent predictors for END. END was significantly associated with poor prognosis at 90 days, and the adjusted OR was 1.74 (95% CI: 1.53–1.97). The incidence of END decreased significantly over time until patients were discharged. A multivariate logistic regression analysis revealed that moderate and severe strokes (OR: 1.49, 95% CI: 1.25–1.79) were more likely to be associated with END compared to mild strokes. The incidence and progression of END were mostly (≈50% to 64%) without a clear cause. However, from a pathophysiological point of view, they can be linked to the extension of the necrotic ischemic territory beyond the primary zone of the penumbra, enlarging the existing ischemic territory, clearly indicating a failure of the microcirculation of the brain tissue due to a failure of collateral channels, with inevitable end-stage postreperfusion damage [11]. The pooled incidence of symptomatic intracerebral hemorrhage as a cause of END was 21.4% (95% CI, 15.6–27.1%), and mild ischemic edema accounted for 14% to 27% of END cases. All these results suggest a high incidence of END in the patient population in the first 24 h after IVT, with a subsequent poor prognosis of the outcome, and put forward the requirement for the effective management of these patients with a focus on END.

In our analysis, we defined END as an increase of four or more points in the NIHSS rather than two or more points because smaller changes in NIHSS might limit the reliability of the results [12]. In our cohort of consecutive acute stroke patients, the incidence of END within 24 h after IVT was 32 (6.5%); the difference in incidence in the two groups of patients (eAT vs. sAT) was nonsignificant (24 (5.8%) vs. 8 (9.8%) *p* = 0.180, OR 0.60). Although nonsignificant, the OR result suggests that eAT has a beneficial effect in lowering the incidence of END. There was also no significant difference in the incidence of END between the two groups in the longer time period between 24 h and 7 days or discharge after AIS, although it was significantly lower (total 17 (3.5%), eAT 12 (2.9%) vs. sAT 5 (6.1%), *p* = 0.147, OR 0.4678). Similarly, despite being nonsignificant, this OR also suggests that eAT has a beneficial effect in lowering the incidence of END after IVT. There is a significant difference between the incidence of END in the first 24 h after stroke in the eAT group compared with the incidence of neurological deterioration between 24 h and 7 days after IVT or at hospital discharge (5.8% vs. 2.9%, *p* = 0.037). In contrast, we did not observe a significant difference in the sAT group (9.8% vs. 6.1% *p* = 0.383). We can see a significant effect of eAT in reducing the incidence of END in the time period between 24 h and 7 days or discharge. Our findings might suggest that eAT could have a neuroprotective effect as it had a significant association with excellent prognosis.

As an efficacy endpoint of eAT after IVT, we chose a 3-month clinical outcome. The data analysis showed that eAT significantly increases the chance of excellent clinical outcome (mRs 0-1). A meta-analysis of randomized controlled trials comparing eAT and sAT after IVT included 1008 patients. The primary efficacy endpoint was a good 90-day prognosis (mRS 0-1 or return to baseline mRS). Compared with routine treatment, eAT did not affect 90-day efficacy (95% CI 0.97–1.32) [13].

As a measure of the effectiveness of eAT compared with sAT post IVT, we chose the functional outcome 3 months after IVT. We did not set a cut-off value for mRS, but we observed the rate of patients at each mRS value and that of patients with excellent and good functional outcomes. In our patient cohort, there was a statistically significant difference in excellent outcome mRS 0-1 in the eAT group compared to patients with sAT after IVT for acute stroke (211, 53.1% vs. 28, 34.6%; *p* = 0.002; OR 0.46; 95% CI: 0.28–0.76).

A multicenter randomized clinical trial, ARTIS, aimed to determine the efficacy and safety of eAT after IVT. Unfortunately, the data analysis revealed a significant safety risk and showed that the intravenous administration of 300 mg of aspirin within 90 min of starting IVT increased the risk of sICH in patients with AIS. In the aspirin group, sICH occurred more frequently than in the standard care group (4.3% vs. 1.6%; 95% CI 0.2–5.4; *p* = 0.04). In addition, sICH was a more frequent cause of poor outcome in the aspirin group compared to the standard care group (11% vs. 1%; *p* = 0.006). The trial also failed to prove the efficacy of eAT with intravenous aspirin; no significant difference was found in the incidence of END and clinical outcome after 3 months. The study was terminated early due to an excess of sICH and no evidence of benefit in the aspirin group [14]. We also chose the incidence of sICH as one of safety endpoints, but based on these data, we also preferred the use of an oral form of AT administered 6 to 16 h after IVT and ruled out intracranial hemorrhage.

Amaro and colleagues published a monocentric study with eAT after IVT. However, they included a heterogeneous group of patients treated either with IVT or with a combination of IVT and MT. Intravenous weight-adjusted unfractionated heparin as well as antiplatelet drugs as a part of subsequent AT were administered to patients treated with MT. The study failed to demonstrate a significant difference in the incidence of bleeding (hemorrhagic transformation of ischemic lesion, intraparenchymal hemorrhage and sICH) between the groups of patients with eAT or sAT. No safety risk was observed in the eAT group of patients compared to the sAT group, and eAT demonstrated significantly better functional outcomes at 90 days post stroke follow-up [15]. We decided to exclude the patients treated with MT to eliminate the risk of bias.

Despite the potential risk of intracranial hemorrhagic complications, eAT may not represent a safety issue. An analysis of data from 24,061 patients from a multicenter registry database demonstrated that patients treated with eAT after IVT for AIS with an already documented hemorrhagic transformation of infarction were more likely to have a favorable outcome (55.7% vs. 39.5%; OR 1.565; *p* = 0.008) and a lower rate of END (12.6% vs. 21.4%; OR 0.585; *p* = 0.009) compared to sAT. Notably, eAT was not significantly associated with an expansion of hematoma or an increased risk of bleeding [16]. After IVT for AIS in our homogeneous group of 489 consecutive patients, no sICH occurred in either the eAT or the sAT group.

In a retrospective observational study evaluating patients with AIS after IVT using eAT compared to patients with sAT, there was no significant difference in the incidence of sICH, the length of hospital stay, in-hospital mortality, gastrointestinal bleeding or adverse patient outcomes [17]. Mortality within 3 months after IVT was our second safety endpoint. There were significantly fewer deaths within 90 days after IVT in the eAT group compared to the sAT group (44, 10.8% vs. 17, 20.7%; *p* = 0.013; OR 0.52; 95% CI: 0.28–0.95). Our data confirm the above results, showing that rational use of eAT after IVT does not represent a risk of harming the patients, does not increase mortality, improves the neurological outcome and reduces the risk of early neurological deterioration.

The main limitations of this work are the monocentric nature of the observational study and the unbalanced nature of the patient sub-sample, especially with regard to the gender of the patients; however, this difference is not significant (57% males). Additionally, the percentage representation of the different stroke etiologies does not reflect the known representation according to TOAST [8].

## 5. Conclusions

Current guidelines for the treatment of acute ischemic stroke do not recommend AT or anticoagulation therapy in the secondary prevention of AIS during the first 24 h after receiving IVT. Due to the short elimination half-life of rt-PA, patients remain unprotected during the critical period where the risk of ischemic stroke recurrence is the highest.

In our prospective monocentric observational cohort study of patients treated with eAT or sAT after IVT for acute stroke, we found that eAT significantly increased the odds of excellent patient outcome 3 months after rt-PA treatment. The patients were not at a higher risk of symptomatic intracranial hemorrhage and had a statistically significantly lower risk of death during the 3-month follow-up period. We observed a reduced risk of END in the eAT group, although the level did not reach statistical significance.

Further randomized controlled trials are needed to evaluate the effectiveness and safety of eAT, in order to find adequate protection against ischemic stroke recurrence without increasing the risk of harming the patient. In addition, larger clinical trials are needed to confirm the data on reducing the risk of END.

## Figures and Tables

**Table 1:** Baseline data.

	Total	Deviation	No Deviation	*p*-Value
n, (%)	489 (100.0%)	407 (83.2%)	82 (16.8%)	-------
Age (mean ± SD; median)	69.94 ± 12.74; 71.00	69.81 ± 13.15; 71.00	70.56 ± 10.47; 72.00	0.517
Men, n (%)	278 (56.9%)	233 (57.2%)	45 (54.9%)	0.702
Women, n (%)	211 (43.1%)	174 (42.8%)	37 (45.1%)	
Arterial hypertension, n (%)	386 (78.9%)	313 (76.9%)	73 (89.0%)	0.014
Diabetes mellitus, n (%)	162 (33.1%)	131 (32.2%)	31 (37.8%)	0.326
Atrial fibrillation, n (%)	81 (16.6%)	63 (15.5%)	18 (22.0%)	0.149
Pre-stroke mRS (mean ± SD; median)	0.54 ± 0.99; 0.00	0.53 ± 0.97; 0.00	0.55 ± 1.07; 0.00	0.867
Baseline NIHSS (mean ± SD; median)	8.19 ± 4.93; 7.00	7.95 ± 4.82; 7.00	9.35 ± 5.33; 8.00	0.019
Systolic BP (average ± SD; median)	159.12 ± 27.44; 160.00	158.07 ± 27.19; 158.50	164.42 ± 28.43; 160.00	0.056
Diastolic BP (average ± SD; median)	85.88 ± 14.93; 85.00	85.10 ± 14.29; 85.00	89.78 ± 17.36; 90.00	0.001
Blood glucose level (mmol/L) (average ± SD; median)	8.27 ± 4.22; 6.91	8.16 ± 4.12; 6.91	8.88 ± 4.60; 7.21	0.158
Blood fibrinogen level (g/L) (average ± SD; median)	3.20 ± 0.90; 3.14	3.15 ± 0.81; 3.07	3.58 ± 1.05; 3.51	0.001
Acetylsalicylic acid, n (%)	152 (31.1%)	126 (31.0%)	26 (31.7%)	0.901
Clopidogrel, n (%)	61 (12.5%)	50 (12.3%)	11 (13.4%)	0.784
LVO + MeVO, n (%)	147 (30.1%)	118 (29.0%)	29 (35.4%)	0.249

**Table 2:** Safety and efficacy data.

	Total	Deviation	No Deviation	*p*-Value
n, (%)	489 (100.0%)	407 (83.2%)	82 (16.8%)	-------
END (4+ NIHSS), n (%)	32 (6.5%)	24 (5.9%)	8 (9.8%)	0.198
END any, n (%)	60 (12.3%)	45 (11.1%)	15 (18.3%)	0.071
Neurological deterioration (24 h–7 d/dis) (4+ NIHSS), n (%)	17 (3.5%)	12 (2.9%)	5 (6.1%)	0.147
Neurological deterioration (24 h–7 d/dis) (any), n (%)	39 (8.0%)	26 (6.4%)	13 (15.9%)	0.004
MAPT	41 (8.4%)	41 (10.1%)	0 (0.0%)	NA
DAPT	366 (74.8%)	366 (89.9%)	0 (0.0%)	NA
Symptomatic intracranial hemorrhage, n (%)	0 (0.0%)	0 (0.0%)	0 (0.0%)	NA
3-month mortality n (%)	61 (12.5%)	44 (10.8%)	17 (20.7%)	0.013

**Table 3:** Three-month functional outcomes.

	Total	Deviation	No Deviation	*p*-Value
n, (%)	478 (100.0%)	397 (83.1%)	81 (16.9%)	-------
mRS 0, n (%)	140 (29.3%)	125 (31.5%)	15 (18.5%)	0.019
mRS 1, n (%)	99 (20.7%)	86 (21.7%)	13 (16.0%)	0.249
mRS 2, n (%)	55 (11.5%)	37 (9.3%)	18 (22.2%)	0.001
mRS 3, n (%)	59 (12.3%)	52 (13.1%)	7 (8.6%)	0.262
mRS 4, n (%)	44 (9.2%)	38 (9.6%)	6 (7.4%)	0.533
mRS 5, n (%)	20 (4.2%)	15 (3.8%)	5 (6.2%)	0.327
mRS 6, n (%)	61 (12.8%)	44 (11.1%)	17 (21.0%)	0.015
mRS 0-1, n (%)	239 (50.0%)	211 (53.1%)	28 (34.6%)	0.002
mRS 0-2, n (%)	294 (61.5%)	248 (62.5%)	46 (56.8%)	0.337

**Table 4:** Etiology of stroke.

	Total	Deviation	No Deviation	*p*-Value
n, (%)	489 (100.0%)	407 (83.2%)	82 (16.8%)	-------
Atherothrombotic, n (%)	253 (51.7%)	216 (53.1%)	37 (45.1%)	0.186
Cardioembolic, n (%)	115 (23.5%)	88 (21.6%)	27 (32.9%)	0.028
Lacunar (n, %)	15 (3.1%)	13 (3.2%)	2 (2.4%)	0.702
Other determined (n, %)	27 (5.5%)	23 (5.7%)	4 (4.9%)	0.773
Undetermined (n, %)	79 (16.2%)	67 (16.5%)	12 (14.6%)	0.670

## Data Availability

The data presented in this study are available on request from the corresponding author due to national legal standards and laws.
